# Honey bees (*Apis mellifera*) preselected for Varroa sensitive hygiene discriminate between live and dead *Varroa destructor* and inanimate objects

**DOI:** 10.1038/s41598-023-37356-x

**Published:** 2023-06-26

**Authors:** Lina Sprau, Kirsten Traynor, Peter Rosenkranz

**Affiliations:** State Institute of Bee Research, University of Hohenheim, Stuttgart, Germany

**Keywords:** Zoology, Animal behaviour, Entomology

## Abstract

*Varroa destructor* is one of the main causes of colony losses of the western honey bee (*Apis mellifera*). Many efforts exist to breed honey bees resistant to *V. destructor*. Varroa sensitive hygiene (VSH) is a commonly selected behavioural trait; VSH workers remove the pupae of mite infested brood cells with high efficiency, interrupting the reproduction of the mite. The cues and triggers for this behaviour are not yet fully understood. To determine what elicits this removal behaviour, we examined preselected VSH workers´ responses to four different groups of objects inserted into freshly capped cells: live mites, dead mites, odour reduced mites, and glass beads. These were also compared to control cells that were opened and closed without inserting any object. The pupae in cells containing inorganic objects (glass beads) were removed at similar rates to the control, demonstrating that an object alone does not trigger a removal response. Dead and odour reduced mites were removed at a higher frequency than control cells, but less frequently than live mites. Workers sometimes removed items resting near the top of the cell without removing the pupa. Our results demonstrate that although mite odour from dead mites triggers removal behaviour, the pupa of cells containing live mites were removed more frequently, suggesting that other cues (i.e. odour from feeding wound) or signals (i.e. pupal movement to signal distress) are important. Future research should focus on elucidating these other cues or signals from the brood and mites, as mite presence alone seems to be insufficient.

## Introduction

Hygienic behaviour in *Apis mellifera* honey bees against various pathogens and parasitic threats is an important social immune response that help protects the colony^1,2^. This behaviour includes the ability to detect, uncap and remove diseased and dead brood^3^. Besides the removal of American Foulbrood (AFB)^4^ and other diseases^5^ a specific hygienic behaviour against *Varroa destructor,* known colloquially as the varroa mite, has been documented^6,7^. This specific removal behaviour toward brood cells infested with mites has been investigated in numerous studies^8–10^ and has been named Varroa sensitive hygiene (VSH)^11^. Honey bees displaying VSH behaviour more readily detect mite infested brood cells and remove the developing honey bee, which consequently inhibits the reproductive cycle of the varroa mite^8,12^.

The varroa mite remains one of the greatest threats to honey bee health and causes widespread colony losses of *A. mellifera*^13^, especially in winter. Breeding and selection programs worldwide seek a sustainable approach to control the mite and are thus attempting to breed varroa tolerant or resistant stock using a variety of different selection criteria^14–16^. Hygienic behaviour towards mite infested brood cells has been identified as a biological method to reduce mite infestation levels in colonies^17^. Hence, VSH behaviour shows great potential to reduce varroa mite pressure within a colony by interrupting the reproductive cycle of the mite^18^. During the brood inspection and removal process the mite is occasionally damaged or killed^19,20^. Even if the cycle is “simply” interrupted and the mite is able to re-enter another cell, this interruption and delay in reproduction can still substantially slow the exponential growth of varroa mites in a colony^17,21,22^. VSH behaviour has been documented as one of the suite of behavioural adaptations in natural surviving colonies, demonstrating the usefulness of this trait to reduce mite loads and improve colony survival^23,24^. VSH has thus been incorporated into many selection and breeding programs that focus on varroa resistance^16,25,26^. Signals originating from the mite such as odour^27,28^ or movement, as well as signals originating from the pupae (damage-dependent removal behaviour^29^ or movement of the pupae) are seen as potential triggers for VSH behaviour.

In addition to the complete removal of the brood from the cell as seen in colonies with high levels of VSH, the uncapping and recapping of cells, known as recapping behaviour (REC), is also an important trait for varroa resistance. When the workers open the cell to inspect it, they could also disturb and interrupt the mite’s reproductive cycle; in some instances the female mite leaves the opened cell without completing her reproductive cycle^15,30,31^. Recapping might represent the first step of the VSH trait and thus high levels of recapping behaviour are also indicators of varroa resistance^24,32^.

The aim of the study was to observe the removal behaviour of workers in colonies selected specifically for their VSH behaviour as well as unselected “wildtype” colonies towards varroa mites with manipulated odour and movement cues. We compared responses to (1) live mites, (2) dead mites, which are thus non-moving, (3) odour reduced mites, which are thus immobile and have a minimal natural mite odour (4) inanimate, inorganic objects (glass beads), and (5) control cells. Control cells were opened and closed in the same manner as was done when inserting an object inside the cell without actually inserting anything. The identical manipulation of all cells was performed to better understand the cues and triggers of removal and recapping behaviour. Is an intrusive, foreign object such as the bead enough of an interference to trigger VSH behaviour and have the workers remove the pupae inside of a cell? Or do special cues like mite movement and feeding or mite odour need to be present to trigger a response? We analysed rates of removal and recapping behaviour in response to the four different treatment groups inserted into a brood cell with appropriately aged larvae compared to control cells. Additionally, we investigated what occurred with the object inside the cell during the eight days of pupal development, documenting the object´s continued presence and position of the object in cells that were not cleared out by the workers.

## Material and methods

The research was conducted in 2021 using 16 colonies managed in Mini Plus hives with approximately 4,000 individuals per colony of *Apis mellifera* Buckfast. The Mini Plus system is a useful tool to simulate a colony in a smaller scale with a reasonable number of worker bees (Mini Plus Styropor ®, 28 × 28 × 26 cm; six frames per hive body, three hive bodies per colony). 13 daughter queens were reared from one VSH colony with high rates of VSH behaviour (51 ± 6 %; details below) and were therefore sister queens. These sister queens were artificially inseminated with brother drones from another VSH selected colony, a typical cross used by breeders selecting for varroa resistance. Approximately 8 µl semen was used per queen, an amount harvested from 8 to12 drones depending on the sperm abundance per drone. The VSH mother was tested for VSH in 2020 in three repeated measurements using artificial infestation of 30 freshly capped brood cells, each infested with a single mite and 30 control cells where only the cell cap was manipulated as in the mite infestation, but nothing was inserted^33^. The average VSH value was a 73 ± 8 % removal rate of mite infested cells. This colony also had a removal rate of control cells of 22 ± 2%, thus giving the mother colony a VSH value corrected for control cell removal of 51 ± 6 %. The drone colony was analysed for VSH in 2019 a single time using the same direct infestation of freshly capped cells and shown to have a mite removal rate of 85 % and removal rate control cells of 4 %; resulting in a corrected VSH value of 81 %. The other three “wildtype” colonies were sister queens reared from unselected stock and open mated as is typical in Germany outside of breeding programs, thus providing a comparative stock typical of honey bees unselected for varroa resistance.

### Treatments

**Figure 1:**
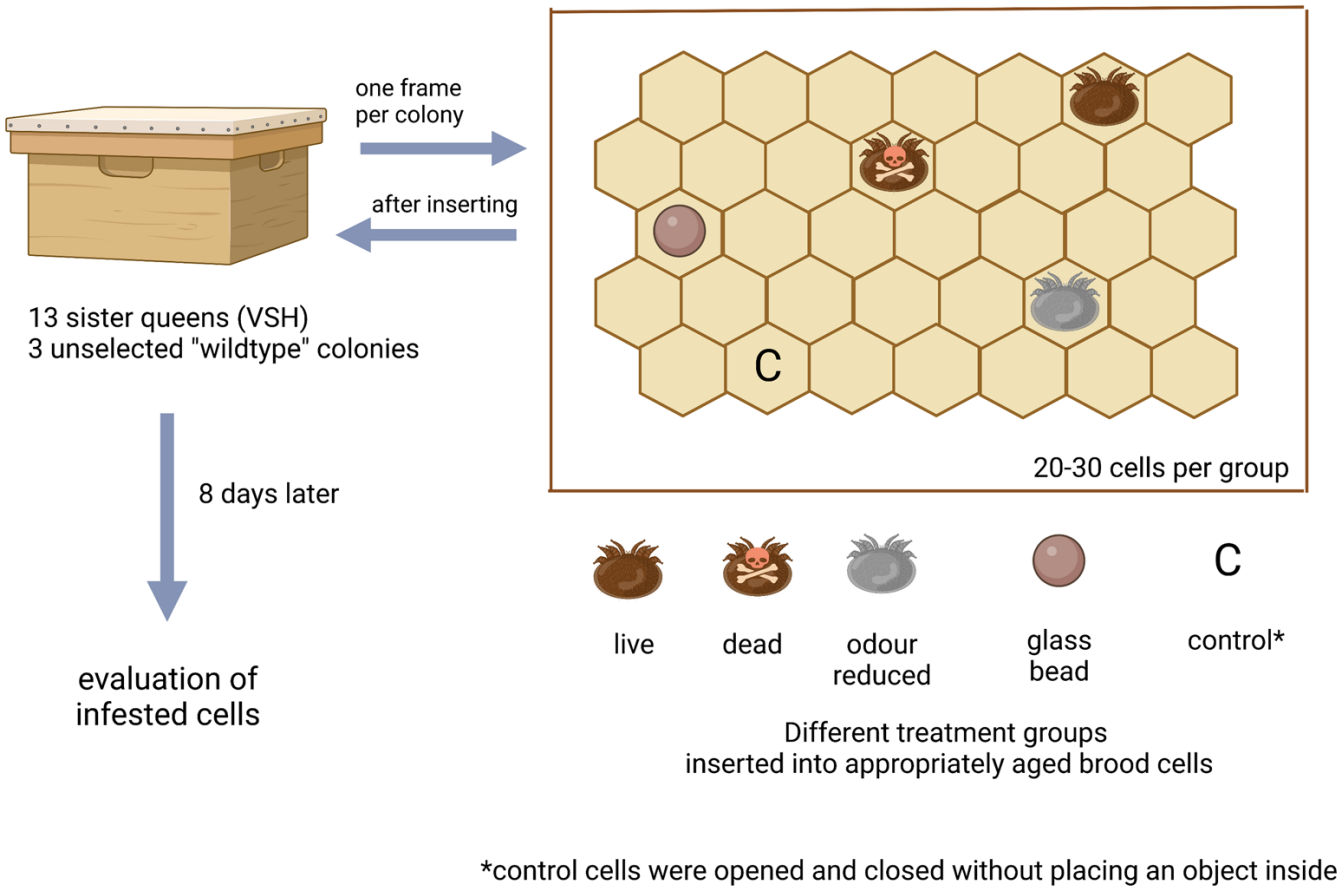
Schematic overview of the experimental procedure. We had five different treatments of live, dead, and odour reduced mites, glass beads, or control cells. In control cells the cell cap was manipulated in an identical fashion, but no object was placed inside the cell. These objects were inserted into freshly capped cells and their location noted. All together we examined removal behaviour in 13 VSH sister colonies and three unselected “wildtype” colonies. After object insertion, the frame was returned to the colony for eight days to evaluate if the bees would engage in hygienic behaviour and remove objects from the cells.

Five treatment groups were formed to determine removal behaviour of the workers in response to various objects placed into appropriately aged freshly capped larval cells (Fig. 1). The groups of objects were: (1) live mite, (2) dead mite, (3) odour reduced mite, (4) odourless glass bead, or (5) a control, where the cell capping was manipulated by cutting a small insertion and then resealing the wax capping as in the other groups, but nothing was placed inside the cell. The objects were placed inside a freshly capped cell less than six hours post capping. Freshly capped cells were chosen so the complete developmental timeline was known and to ensure that the pupae had not yet spun her cocoon. The mites or objects were inserted inside the cell by making a small incision in the cell cap, then placing the mite or object inside of the cell on the dorsal side of the larvae, and resealing the cell cap gently (20–30 cells per group). The cells to be infested were randomly chosen on both sides of a brood comb and the location of inserted objects was marked on a transparent sheet using different markings for each treatment group.

### Varroa mites

The mites were harvested using the powder sugar shake method^34^. Live mites were collected and inserted into appropriately aged brood cells on the same day. Dead mites and odour reduced mites were collected one month prior to the experiment. Dead mites were immediately frozen after collection and stored at −20 °C. Odour reduced mites were stored for one month in pentane, to reduce the CHC molecules on the outside of the mite^35^, then air dried to allow the pentane to evaporate. The glass beads (Rocailles; colour: topaz) were approx. 1.5 mm in diameter, comparable to the size of a mother mite.

### Evaluating worker response to cells

After inserting the objects inside of the cell, the brood frame was returned to the host colony. Eight days later the brood comb was retrieved from the colony for the evaluation. The evaluation of the brood comb was conducted by documenting the number of cleared out cells, the cell recapping status, and the current placement of the inserted object.

### Experimental design and evaluation

The procedure of inserting objects into appropriately aged brood cells was repeated one to four times per colony. In the first two rounds the experiments were conducted with three groups (live mites, dead mites, and control cells). The subsequent two rounds were designed with all five groups. In two out of the 16 colonies no beads or odour reduced mites were inserted. The removal rate is calculated by dividing the amount of cleaned out cells by the total amount of infested cells.

The placement of the object was categorized into two zones: Top and Bottom. Top included the region on and around the honey bee’s thorax, head or the cell cap. Bottom included the honey bee’s abdomen or the bottom of the cell. In addition to the five treatment groups, 50 additional cells on one brood comb were infested with dead mites and beads. This frame was not returned to the hive, but placed in a brood chamber for eight days, so the workers could not access the cells. Subsequently we could determine the position of the object to understand how it might shift when it was only subjected to pupal movement inside the cell. Evaluation of object location took place on the 8th day after inserting the objects, an identical time period as when the frames were returned to the colony and then removed and inspected on the 8th day. The position of the objects was documented to determine if pupal movement alone can cause displacement.

### Recapping behaviour (REC)

Recapping activity was documented by inspecting the cell cap of all remaining closed brood cells where the pupa was still intact and examining it for the presence of the cocoon spun into the cap. The pupae complete the spinning of the cocoon 24 h after cell capping^36^ and therefore the cell cap incision we made within six hours of cell capping to insert the objects has no effect on the documented REC activity. However, if the workers remove the cell cap to inspect a cell and then recap the cell instead of removing the pupa more than one day after we returned the manipulated frame to the colony, then the cocoon threads are missing in the cell capping, evidence that the cell was recapped by the workers^15,37^. The recapping rate is then calculated by dividing the number of recapped cells by the total amount of manipulated cells that still had a cell capping.

### Statistics

All statistical analysis was conducted with the software jmp, version 17.0.0 except for the generalized linear models which were calculated in R 4.2.1. Overall comparisons of removal rates between treatment groups were done via a Kruskal–Wallis test. The comparison of the different groups for brood removal, REC, and missing objects was analysed by a generalized linear model (glm).

The glm was designed with brood removal, REC or the missing objects as variables, the treatment groups as fixed effects and the colony and the repetitions as random effects.

The model reads as follows: glmer (cbind (open, closed) ~ group + (1 | colony/replicate), family = binomial). By using the command cbind the different number of inserted cells could be included into the analysis. The residuals of each model (brood removal, REC and missing object) were normally distributed.

The data in the graphs are displayed as rates. Removal rate describes the number of cells where the cell content was removed compared to the number of infested cells for each group. The recapping rate is based on the cells displaying signs of recapping divided by the remaining closed cells. We also defined the rate of objects missing by dividing the number of missing objects by the number of remaining closed cells. To compare the position of the different groups a Chi^2^ test was performed.

## Results

**Figure 2:**
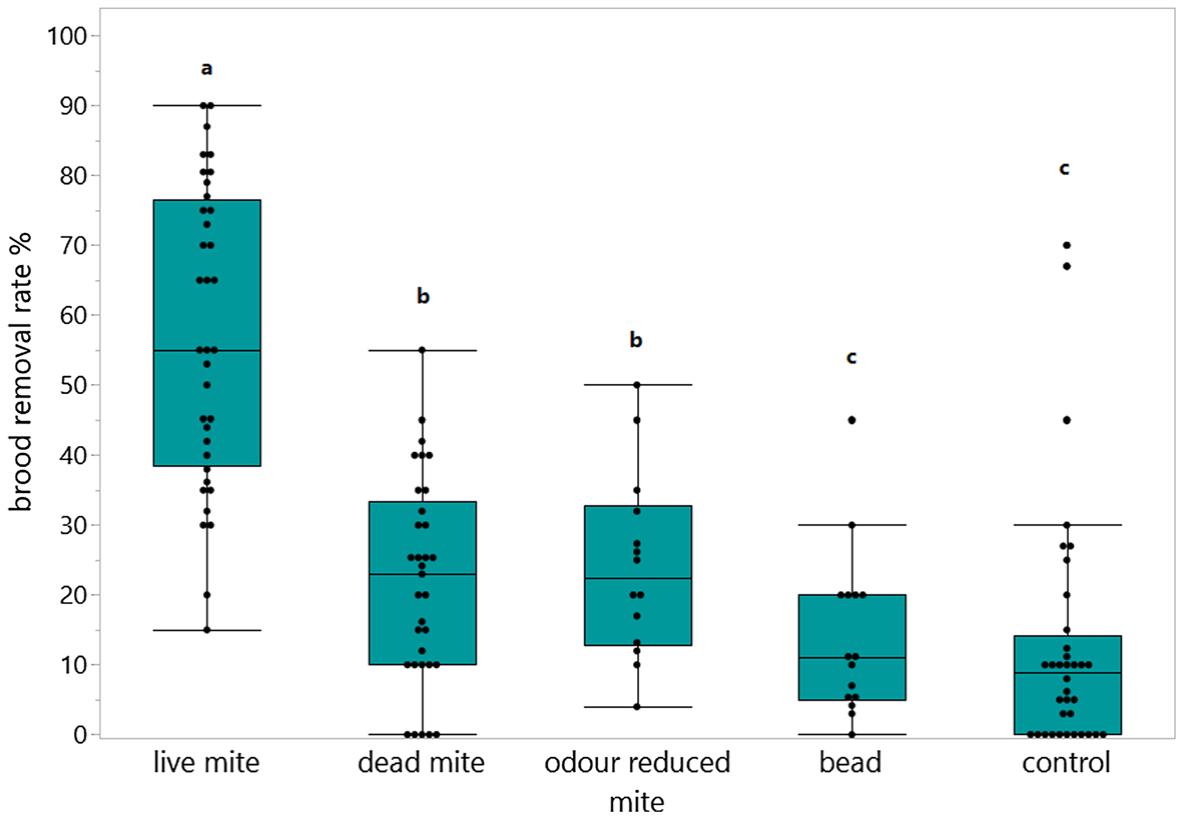
Brood removal behaviour for each treatment group: live mite (n = 915 cells inserted); dead mite (n = 798 cells); odour reduced mite (n = 407 cells); bead (n = 420 cells); control cells (n = 915 cells). The brood removal rates were calculated by dividing the number of removed cells by the number of infested cells. The removal data were compared using a generalized linear model (glm). Significant differences are indicated by different letters (*p* < 0.01), for detailed *p*-values, see Table S1. Data from 16 colonies; 13 VSH and 3 unselected “wildtype” colonies. The points show the values of every measurement independent of the colony.

We compared the removal rates across five treatment groups: live mites, dead mites, odour reduced mites, glass beads, and control cells in a total of 3456 manipulated cells and they differed significantly between the groups (glm, *p* < 0.0001, n = 16 colonies (repeated between 1 and 4 times); Fig. 2). Regardless of VSH selection background (VSH stock vs. unselected “wildtype”), all colonies removed significantly more pupae of cells containing live mites (mean = 56.2 ± 21.4 %) compared to dead (mean = 21.2 ± 14.8 %) and odour reduced mites (mean = 21.6 ± 13.4 %). These two groups of immobile mites were cleaned out of the cells at a significantly higher rate than the beads (mean = 12.4 ± 11.8 %) or the control cells (mean = 12.2 ± 16.7 %). When comparing the wildtype to the selected colonies, the removal rate of the VSH selected colonies (mean = 28.2 ± 24.7 %) showed a higher overall removal rate than the unselected colonies (mean = 15.6 ± 18.3 %) (Fig. S1), however due to high variance in the measurements no significant difference between both groups exists (Kruskal–Wallis Test, df = 4, *p* > 0.05, n = 16 colonies (repeated 1–4 times per colony)).

**Figure 3:**
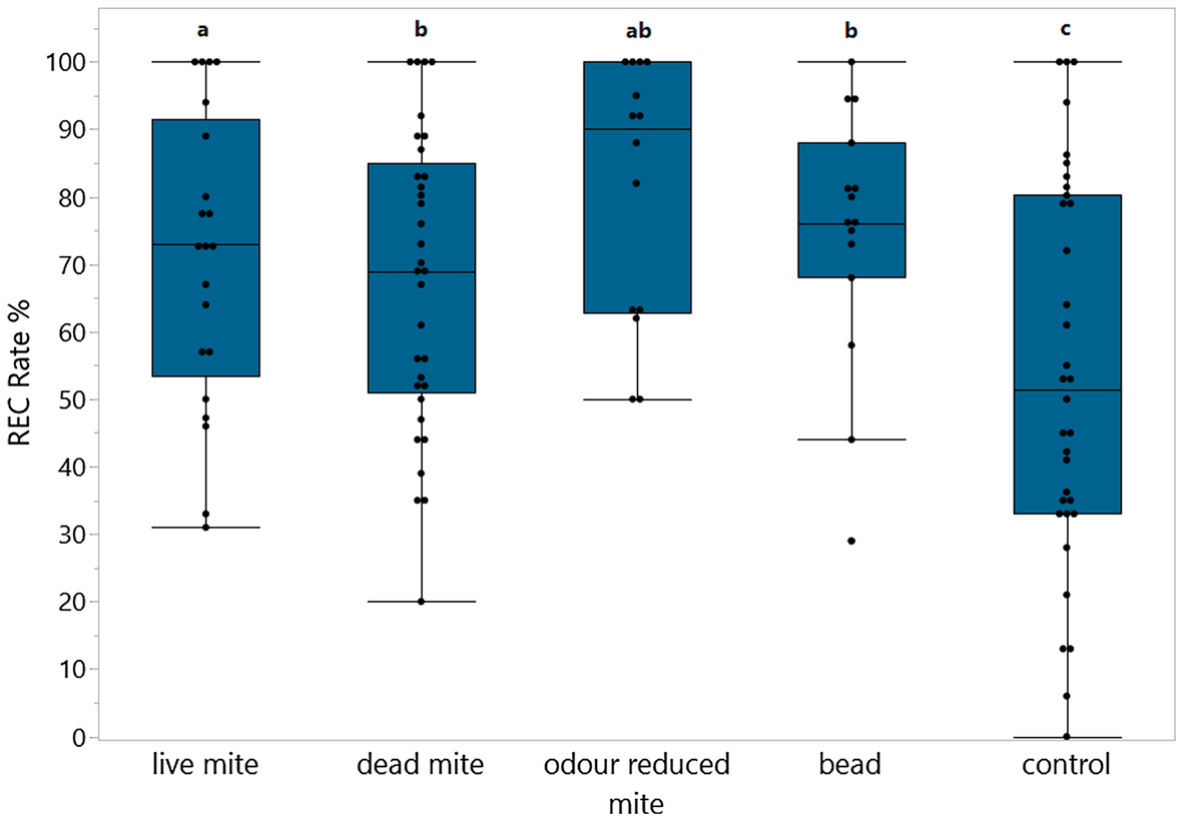
Recapping rates of cells that were capped at time of frame evaluation eight days post insertion. We calculated the recapping rates (recapped cells/ all capped cells) for the five treatment groups: live mite (n = 323 capped cells); dead mite (n = 630 capped cells); odour reduced mite (n = 332 capped cells); bead (n = 373 capped cells); control cells (n = 777 capped cells). The REC data were compared via a glm. Different significance levels are indicated by different letters (*p* < 0.01; Tab. S1). Since the “wildtype” colonies had significantly lower rates of recapping, this figure only shows the recapping rates of the 13 VSH daughter colonies. For the comparison of VSH and unselected colonies see Fig S.2. The points show the values of every measurement independent of the colony.

In total 2516 cells were capped eight days post insertion (72.8 % of all manipulated cells) and could thus be examined for recapping and to determine if the inserted objects had been removed (Table 1). At least eight manipulated cells that were capped had to be present at the evaluation time for the treatment to be included in the REC analysis (n = 17 of the 134 values of treatments groups had to be excluded). The recapping behaviour differed significantly across the treatment groups among the 13 colonies selected for VSH and was quite high across the board (glm, *p* < 0.001, n = 13 colonies (repeated 1–4 times per colony): live mite: mean = 66.2 ± 25.3 %, dead mite = 64.0 ± 24.5 %, odour reduced mite = 72.8 ± 27.3 %, bead = 65.6 ± 27.8 % and control cells = 51.5 ± 29.0 % (Fig. 3). The unselected colonies had a significantly lower recapping rate than the VSH Colonies (Kruskal–Wallis, df = 4, *p* < 0.001, n = 16 colonies (repeated 1–4 times per colony): see Fig. S2).

**Table 1:** Overview of the number of infested cells, removed brood, recapped and missing objects per treatment group as well as the different rates (removal rate, REC in capped cells, REC in all cells). In the removal rate and missing objects all colonies were considered, the REC data is only based on the VSH colonies where at least eight cells were capped at time of evaluation.

Groups	Infested cells	Brood in cells removed	% Removal rate (mean ± SD)	Cells still capped	Cells with evidence of recapping	% Recapped of capped cells (mean ± SD)	% Recapped of all cells (mean ± SD)	Total number of objects in capped cells	Missing objects total	Missing objects with recapped cells	Missing objects without recapped cells
Data from	All colonies			Only REC considered*				All colonies			
Live mite	916	528	56.2 ± 21.4	323	202	66.2 ± 25.3	32.1 ± 21.6	388	85	60	25
Dead mite	798	170	21.2 ± 14.8	630	383	64.0 ± 24.5	48.8 ± 17.2	628	131	90	41
Odour reduced mite	407	82	21.6 ± 13.4	332	227	72.8 ± 27.8	57.1 ± 23.5	325	83	65	18
Bead	420	48	12.4 ± 11.8	373	232	65.6 ± 27.8	56.3 ± 23.7	372	35	28	7
Control cells	915	112	12.2 ± 16.8	777	400	51.5 ± 27.8	44.5 ± 23.7	–	–	–	–
Total	3456	940	27 ± 24.4	2435	1444	62.3 ± 27.3	46.1 ± 23	1713	334	243	91

*Recapping measurements only taken in VSH colonies, when at least 8 capped cells were available at time of evaluation. Colonies that had cleaned out more cells were excluded.

**Figure 4:**
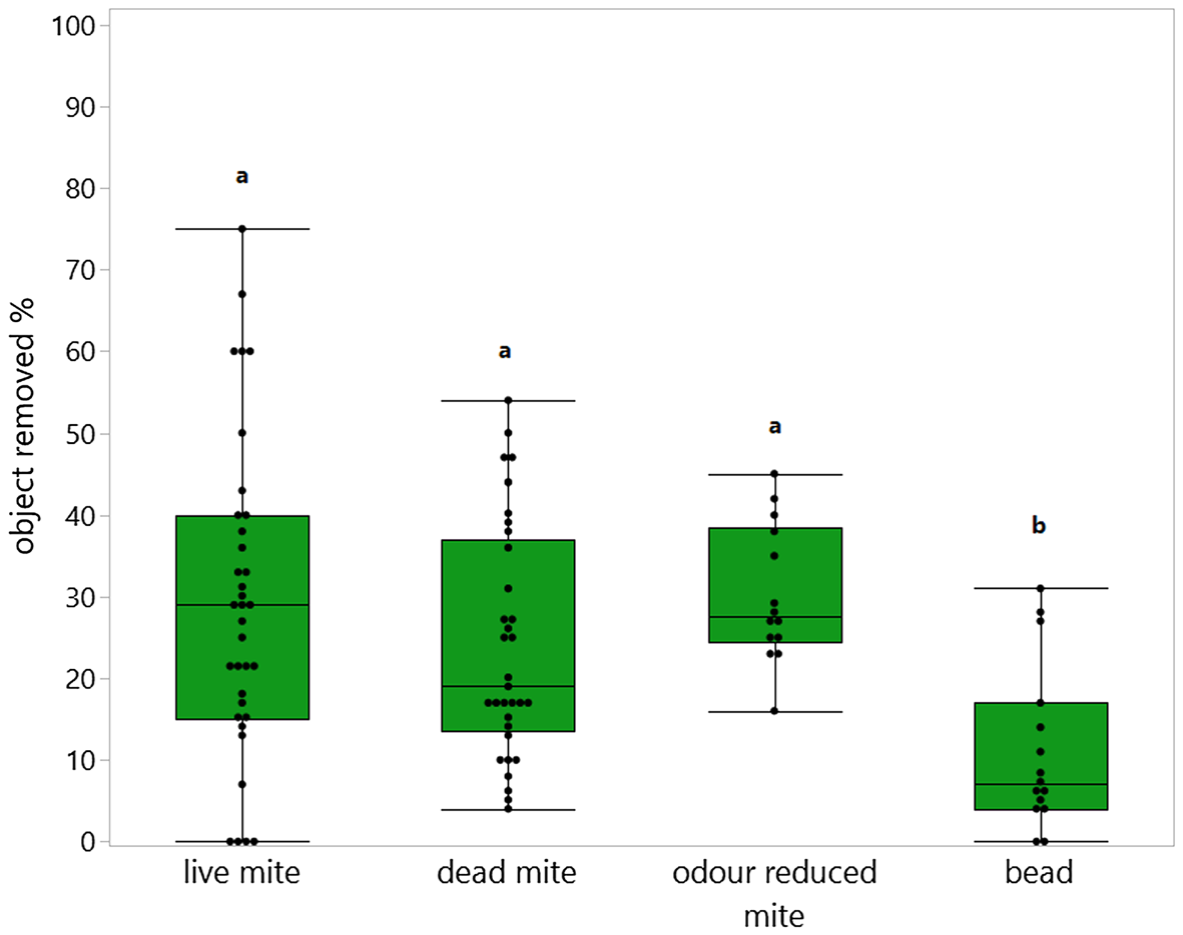
Object removal from capped cells. We calculated the rates at which objects in capped cells were removed by VSH selected stock (number of cells where the object was removed/ total capped cells at the time of evaluation) for the five treatment groups: live mite (n = 388 cells); dead mite (n = 628 cells); odour reduced mite (n = 325 cells); bead (n = 372 cells). The rates were compared via a glm (Table S1). Different significance levels (*p* < 0.01) are indicated by different letters. The points show the values of every measurement independent of the colony.

Regardless of recapping status, in total 22.9 ± 16.5 % of objects were missing from cells that were still capped eight days post cell manipulation: live mite (27.0 ± 20.0 %), dead mite (22.9 ± 14.1 %), odour reduced mite (27.2 ± 11.2 %) and bead (10.2 ± 9.6 %). The treatment groups with mites were missing from the cells more often than the beads (glm, *p* < 0.001, n = 13 colonies (repeated 1–4 times per colony, Fig. 4).

When we look at the cells still capped in all colonies at time of frame evaluation, the greatest proportion had been recapped, but still had the object inside (n = 853), followed by cells the workers ignored and never inspected, as they lacked recapping and still had the object inside (n = 526). Cells that were recapped after the workers had removed the inserted object were less frequent (n = 243). In 91 cells, the objects were removed almost immediately after the frame was returned to the colony, as they lacked evidence of recapping and were missing the inserted object.

Of the 1325 objects still found in the cell of all colonies (excluding the group of live mites), 217 objects were found spun into the cocoon or were worked into the cell cap. The remaining 1108 objects were freely lying in the cell or found on the pupae.

**Figure 5:**
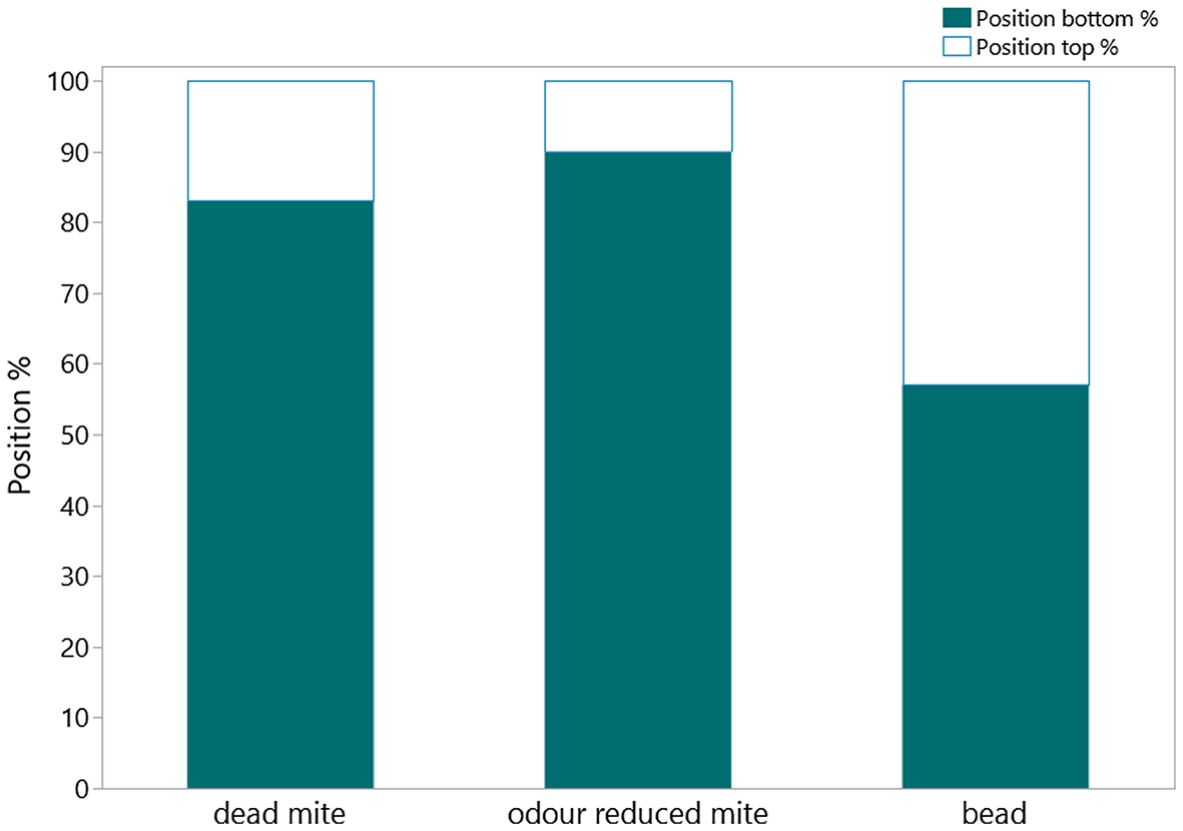
Position of the objects found segregated by treatment group: dead mite (n = 497), odour reduced mite (n = 242) and glass bead (n = 338). The dead and odour reduced mites were mostly found at the bottom of the cell, whereas the bead was also found on the top of the cell in 43.7 % cases (in 146 out of 338).

The objects that remained in the cell were most often found in the bottom half of the cell in all groups, which was expected, because the removal of objects in the lower part of the cell is more difficult unless the entire cell is cleared of its contents including the pupa (Fig. 5). The bead was found on the top of the cell (n = 146) more often than the other two groups. 56 ± 1.9 % of the cells in which the objects were found on top also displayed REC activity.

The experiment to test where objects end up when worker interaction is removed demonstrated that the majority of the dead mites were found on the bottom of the cell (52 %, n = 26), followed by the top (36 %, n = 18), while six could not be counted due to the death of the pupae (n = 5) or it was spun into the cocoon (n = 1). The beads were found predominantly at the top (54 %, n = 27), with a smaller proportion at the bottom (42 %, n = 21), while two (4 %) could not be included as the pupae died in those two cells.

## Discussion

We investigated the behaviour of honey bees in daughter colonies from VSH selected stock to determine their response to varroa mites, where some of the potential cues used for detection and removal of the mite such as movement, pupal feeding or odour were eliminated. We used the direct infestation method to study the removal response, as this method has frequently been used in other studies^33,34,38,39^, and allows the measurement of behavioural response to infested cells with minimal interference to normal worker behaviour. The small incision in the capping can trigger removal behaviour, which is why we always include control cells, where the cells were opened as if an object would be placed inside the cell and then resealed. This direct infestation method allows us to measure the brood removal response to identically manipulated cells into which phoretic mites are inserted, or as in this experiment, other objects.

Despite the fact that we inserted dead mites and dead mites that additionally had their odour reduced, we demonstrate that the workers still recognized the mites and were more likely to remove the pupae than when cells were infested with inanimate beads of similar size. The presence of a foreign object alone is thus not enough of a cue to trigger removal behaviour in either VSH colonies or unselected stock, as even in this unselected “wildtype” stock, the removal rate for all mite groups was significantly higher than for the glass beads (Fig. S1). The removal of pupae containing inorganic objects was similar to the removal of the pupae in control cells. The removal rate of the control cells averaged approximately 12 % and were therefore within an expected range for this method; other studies show similar removal rates in the control cells^7,40,41^. As the cells were randomly chosen, a complete absence of mites cannot be guaranteed and could therefore explain the removal of some of the pupae in the control cells. Notably, cells containing live mites were found to be cleared out most frequently, but the pupae of immobile (dead mites) and odour reduced mites were still removed more frequently than inanimate, inorganic glass beads or control cells, suggesting that the odour and movement of the mite alone are not solely responsible for triggering removal behaviour. Additional factors must be involved in the triggering of removal behaviour. A four-week treatment with pentane may not result in a complete loss of odour. However, pentane is a potent solvent that is capable of reducing compounds associated with mite removal^27^. Our results confirm and highlight the complexity of mite recognition and the cascade of ensuing honey bee behaviours. Movement and odour of the mites^27,28^ are important drivers for hygienic behaviour, but are not the only cue, as otherwise these two groups of manipulated mites would have been removed at identical rates as the control cells. Our results highlight that odour and movement may be less important than previously postulated^27,28^.

Cells that contained dead or odour reduced mites lack the feeding sites on pupae, the potentially spilled pupal hemolymph and fat body, as well as the communal mite faeces used as a gathering point for mite offspring^42^. These are all cues that could trigger the higher removal behaviour demonstrated by workers to the live mites. Another potential route of signalling is from the brood itself, which could recognize the difference in the infesting object or experience harm from the parasite and intentionally or unintentionally signal its caregivers. Cues from brood that trigger removal behaviour have been previously shown for factors such as brood damages caused by virulent virus infections vectored by mites, which trigger a a higher removal rate than uninfected brood^29^ or chemical cues linking *V. destructor* and Deformed wing virus^43^. Different chemical cues originating from the brood or changed brood movements could also trigger removal behaviour^44^. Interestingly, we saw high recapping rates in all treatment groups indicating that a foreign object may trigger cell inspection, but upon viewing the cell contents, the workers recap the cell without removing the pupa.

Older publications in which dead mites were also inserted into cells showed a less pronounced removal of the dead mite^35^ compared to this study or a similar study performed 2021^32^. This is not surprising as we used colonies selected for a specific removal behaviour. This indicates that selective breeding has positive effects and also highlights the importance of continued research and work on breeding varroa resistant honey bees.

In previous studies, cells which were artificially infested with mites sometimes did not contain a mite at the time of evaluation^33^. Was the mite actively removed by the workers or did the mite walk out of the cell during a brief interlude when the cell was uncapped? If the missing mites can be explained solely by their active walking out of the cell, then all inanimate objects should have remained inside of the cells after the period of eight days. However, our results showed that in approx. 31 % of capped cells which show signs of REC activity the mite or object was removed without removing the pupae. The removal rate was significantly higher for all mite groups than the inorganic object (glass bead). The highest variation was found in the group of live mites, indicating a possible combination of the mite actively walking out of the cell and removal by workers if reachable (in extreme cases up to 60 % of the mites were missing). It is conceivable that the mite exits the cell prior to investing a lot of energy in reproduction and therefore exits shortly after being placed inside of the cells as soon as an opportunity presents itself by the cell being uncapped.

A revision of the evaluation criteria for Varroa sensitive hygiene (VSH) may be warranted, as cells in which the mite is not present at the time of evaluation should not be considered in the evaluation of the VSH value. The absence of a mite in a previously infested cell that also shows signs of recapping may indicate that the workers initially detected the presence of the mite in the cell, opened the cell, but then the mite was no longer present and so the pupae was not removed. As a result, the workers may have chosen not to remove the infested larvae or pupae. The bees in such cases thus exhibit a specificity in that they initially detected the mite's presence in the cell, but they conserved energy by not removing the larva when the mite either left or was removed from the cell. This is a desirable trait for which breeders should select. When the mite leaves the cell prematurely, it is unable to complete its reproductive cycle, making removal of the larva unnecessary. However, if REC activity was not documented, there exist the possibility that the mite left the cell through an incomplete resealing of the manipulated cell capping due to improper working, a possibility that cannot be completely ruled out, and thus no adjustment to the VSH score should be made.

When the workers had access to the manipulated brood frame, the dead and odour reduced mites were mostly found in the bottom part of the cell, suggesting that if these mites had been reachable from the top of the cell without removing the pupa, they would have been actively removed by the workers. In contrast the beads were found more often in the top part of the cell compared to the other groups. In some cases, the bead was even incorporated into the wax capping of the recapped cell, demonstrating that the workers used it as building material instead of recognizing it as a potentially harmful object that had to be removed. Our experiment on the distribution of these same objects when interaction with workers was eliminated by placing the manipulated brood frame in an incubator demonstrated that dead mites tend to be moved toward the bottom of the cell during the pupal development phase (52 %), potentially due to cocoon spinning or pupal movement during metamorphosis. In these unattended brood cells, 36 % of the mites could still be found near the top half of the cell, a stark contrast to the dead mite location in brood frames attended by workers, where only 83 out of 497 dead mites (16.7 %) were found near the top, again suggesting that workers actively remove mites if they can be reached. There was not a large difference between the location of the bead on frames of brood without worker attendance (bottom = 42 % vs top = 54 %).

The opening of the cell and the removal of brood are separate traits in a graded response exhibited by adult workers toward mite infested brood cells^39^. REC was high in all treatment groups except in the control cells which indicates a lower threshold for the initial cell opening and inspection of the brood. The brood may signal a general disruptive factor through the cell capping to its caregivers without differentiating between harmless (bead) or potentially dangerous (mite) disturbance, which then triggers the opening of the cell. After removing the cell cap the worker can better assess actual risk and if deemed harmless like the bead, the cell is closed up again. The workers even seem to conserve energy by leaving the harmless object in the cell or incorporating it into the cell capping. Interestingly the number of cells still containing a mite or object which were recapped was higher than the uninspected cells which retained their original wax capping. Moreover, it shows a lower threshold for triggering recapping behaviour than the removal of the brood, which is to be expected, as the energetic cost of recapping is much lower compared to the removal and cleaning of an entire cell, plus the loss of the developing pupa. The initial disturbance signal originating from the pupa could therefore be in response to small disturbances and trigger the worker to uncap the cell. Our results support the statement that recapping plays an important role in the detection of infested brood for removal, but that the cell inspection is not what delays mite reproduction^32,45^.

Cocoon spinning is completed approximately 24 h after cell capping^36^; therefore if cells are opened immediately upon the brood frame being returned to the colony and objects removed, then recapped before the cocoon spinning is completed, we would not be able to document this rapid recapping. The evidence of cells with normal caps and missing objects (n = 91) suggests that some cells are very rapidly inspected and recapped, although these cells make up a tiny proportion (2.6 %) of the cells we manipulated. Such rapid worker responses to brood cells are fascinating and should be kept in mind for future studies.

Our results indicate that the removal behaviour is not triggered by a single factor but seems to be a combination of different cues and signals. Furthermore, our work shows that in daughter colonies of stock selected for VSH, the removal of mite specific cells was elevated compared to older studies, suggesting that breeding and selection efforts are enhancing this trait. Recapping in these VSH stock colonies independently of the treatment groups was very high, suggesting that cell inspection as a form of risk assessment is a common behaviour in honey bees prior to the more drastic step of brood removal. Our studies have shown that workers exhibit active removal of foreign objects from cells if those objects are deemed a hindrance and are within reach during the time of cell inspection. Our results highlight the need for a more detailed understanding of the brood signals that trigger cell inspection by their caregivers and what assessment criteria these workers use to judge if the pupa should be removed or recapped. As olfactory cues appear to play a subordinate role, further studies should focus on factors originating from the pupae, such as damage to the pupae including weight loss, developmental disabilities, and viral infections.

## Supplementary Information

Supplementary Information.

## Data Availability

The datasets generated during and/or analysed during the current study are available from the corresponding author on reasonable request.
