# Supplementary material for: Honey bees (Apis mellifera) preselected for Varroa sensitive hygiene discriminate between live and dead Varroa destructor and inanimate objects
Source: Sci Rep. 2023 Jun 26;13:10340. doi: 10.1038/s41598-023-37356-x (PMC10293197; doi:10.1038/s41598-023-37356-x)
Supplement: Supplementary file 1 — Supplementary Information. [file 41598_2023_37356_MOESM1_ESM.docx]

Supplemental material

Tab. S1 Significance levels between the different categories in the removal rates, recapping rates (capped cells) and removed objects. To analyse the data the variables (removal rates, recapping rates and object removed) were calculated with a generalized linear model (glm).

| Categories | Removal rates | Recapping rates | Object removed |
| --- | --- | --- | --- |
| live mite – dead mite | p<0.001*** | p<0.001*** | p>0.05 (n.s) |
| live mite – odour reduced mite | p<0.001*** | p>0.05 (n.s) | p>0.05 (n.s) |
| live mite – bead | p<0.001*** | p=0.004723 ** | p<0.001*** |
| live mite - control | p<0.001*** | p<0.001*** | ------------ |
| dead mite – odour reduced mite | p>0.05 (n.s) | p=0.048771* | p=0.0234* |
| dead mite - bead | p<0.001*** | p>0.05 (n.s) | p<0.001*** |
| dead mite - control | p<0.001*** | p<0.001*** | ------------ |
| odour reduced mite – bead | p=0.0015 ** | p>0.05 (n.s) | p<0.001*** |
| odour reduced - control | p<0.001*** | p<0.001*** | ------------ |
| bead – control | p>0.05 (n.s) | p<0.001*** | ------------ |


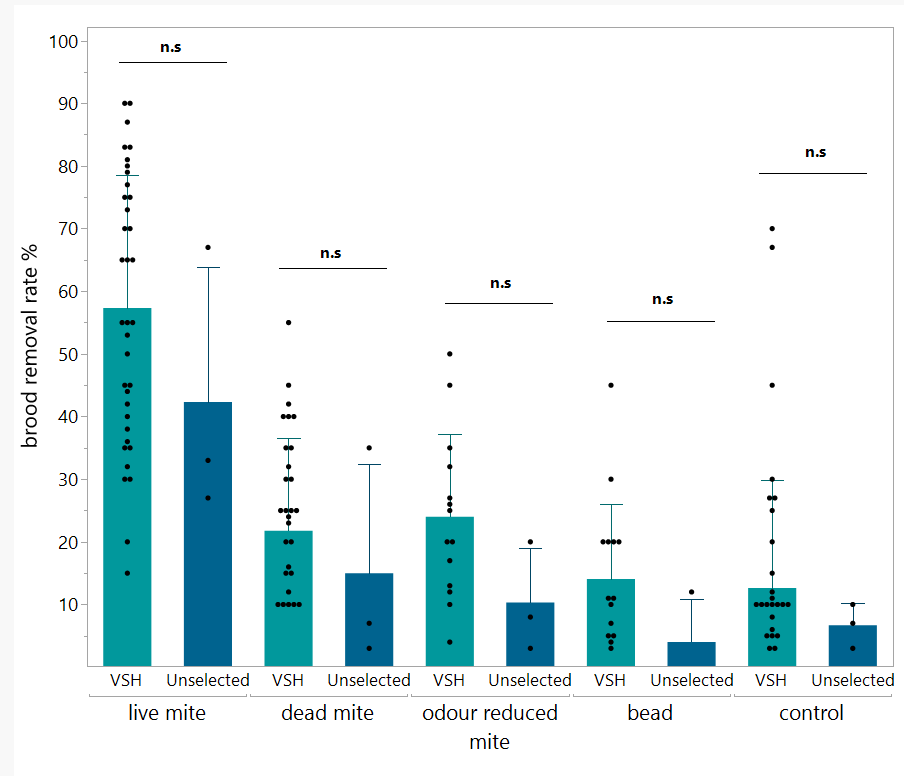


Fig. S. 1 Displayed are the removal rates of the different treatment groups segregated into the VSH colonies and unselected colonies. Only three control stock colonies were tested on a single occasion, compared to 13 VSH colonies that were measured up to four times each. Due to high variability, the control and VSH colonies did not differ significantly (Kruskal-Wallis, n.s = p > 0.05, n = 16 colonies (repeated 1-4 times per colony).


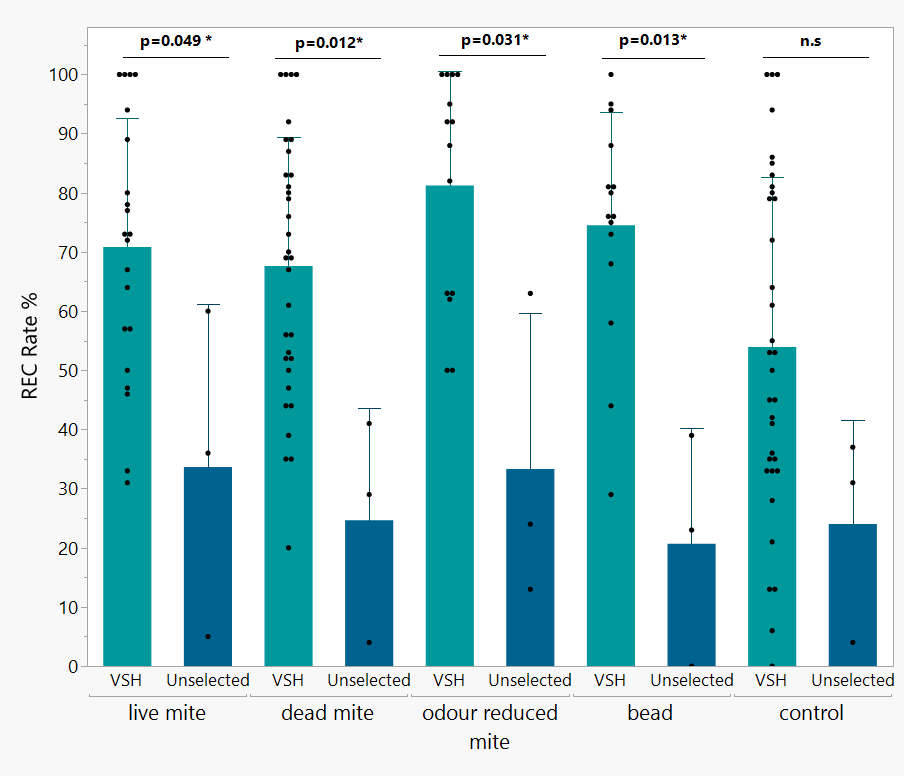


Fig. S. 2 The REC rates of the different treatment groups segregated into VSH colonies and unselected control colonies. The REC rates were significantly higher in selected colonies compared to unselected colonies for all categories except for the control cells, where only the cap was manipulated, but no object or mite was inserted. (Kruskal-Wallis, n.s = p > 0.05, n= 16 colonies (repeated 1-4 times per colony)


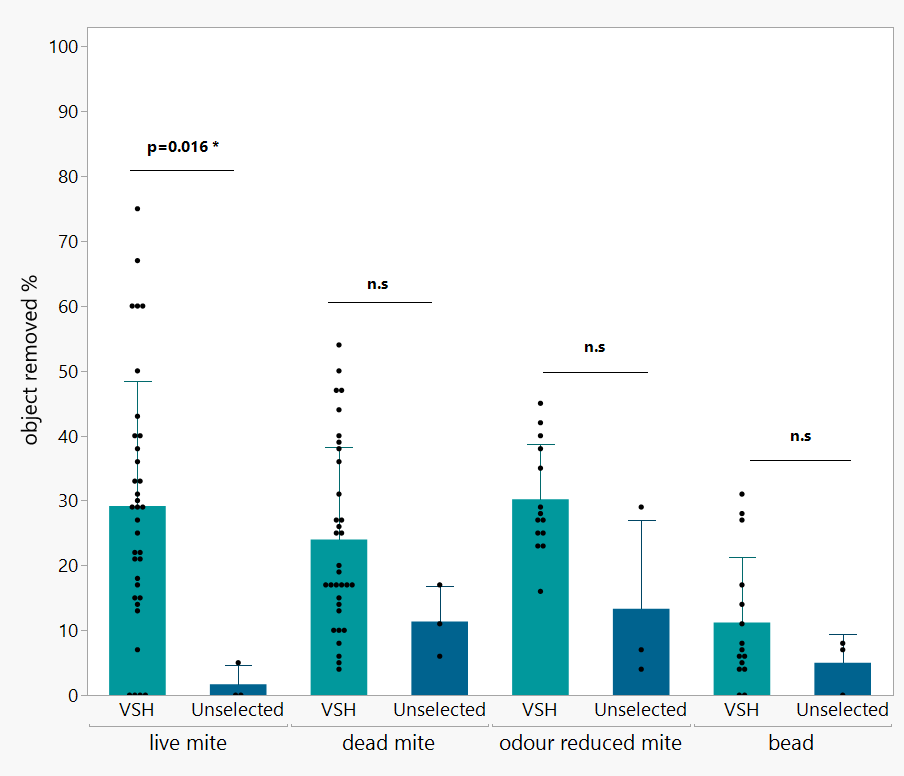


Fig. S. 3 Displayed are the object removed rates of the different categories divided in the VSH colonies and unselected colonies. The rates between the unselected and selected colonies were analysed with a Kruskal-Wallis, n.s = p > 0.05, n= 16 colonies (repeated 1-4 times per colony). As no object was placed inside the cell in the control group this group was not displayed in this figure.
